# Regional culture: The role of the invisible hand in shaping local family firms’ top management team

**DOI:** 10.3389/fpsyg.2022.781744

**Published:** 2022-11-17

**Authors:** Xiaodong Yu, Yuyin Zhang, Xirong Cheng, Huan Li, Yujie Chen, Weizhong Zhou

**Affiliations:** ^1^Business School, Central University of Finance and Economics, Beijing, China; ^2^School of Economics, Beijing Technology and Business University, Beijing, China

**Keywords:** clan culture, family firms, family involvement, executive compensation, socio-emotional wealth, evolutionary psychology

## Abstract

Research on family businesses has focused on the differences between family and non-family firms regarding the top management team (TMT), while this study further explores the difference within family firms from different regional culture based on the perspective of socio-emotional wealth (SEW) and evolutionary psychology. Using a sample comprised of all 625 family firms listed on the Small & Medium Enterprise Board and Growth Enterprise Board in Shenzhen Stock Exchange, this study finds that in regions of strong clan culture, family members are more willing to be involved in a family business and accept lower economic rewards. Particularly, when financial risk is high, these relationships mentioned above become more significant. Based on the results, this study proves that regional culture can affect the characteristics of top management teams in family firms, explains the heterogeneity of family firms’ decision-making from a culture-based perspective and extends existing research on family business from the level of “family firm vs. non-family firm” to that of “family firms affected by different regional cultures”.

## Introduction

Existing studies have focused on the differences between family and non-family firms in terms of the top management team (TMT; De Massis et al., 2021). Compared with non-family firms, as family firms are more concerned with special business goals such as maintaining family control, satisfying altruism, enhancing family reputation, and expanding social networks (Zellweger et al., 2019; Le Breton-Miller and Miller, 2020), they prioritize the interests of the family and family members in both the design of the team structure and the selection of team members (Becerra et al., 2020). For family members, by virtue of their family identity, they can enter the top management team and even play important roles such as Chief Executive Officer (CEO) and Chief Financial Officer (CFO) more easily (Basco et al., 2019). Even if they do not show sufficient management talent, they can still receive firmer support and trust from the firm (Chrisman and Patel, 2012). It has been found that the tenure of family CEOs is three times that of non-family CEOs and that non-family CEOs are twice as likely to be fired when compared to family CEO (Detienne and Chirico, 2013).

However, family firms are not an entirely homogeneous group, and different family firms have different preference degree for family members (Daspit et al., 2021). An important feature that distinguishes family firms from non-family firms is that family members with kinship ties participate in the operation of the business. Therefore, the operation and management of family firms, and the maintenance and coordination of internal relations depend not only on the rules and regulations of the firm, but also on the local cultural concepts (Wang et al., 2022). However, it should not be ignored that the cultural concepts of different regions vary greatly (Zhang, 2020). If family firms have more imprinted with regional culture than non-family firms, do family firms with different cultural imprints exhibit different characteristics? In other words, what is the impact of regional culture where family firm is located on business operations? Does it act as an “invisible hand” that exerts a subtle influence on a series of business behaviors such as the selection of top management team members? No attention has been paid to this topic in existing studies.

Therefore, in order to answer the questions above, this study investigated whether regional culture affects the TMT’s characteristics in family firms according to the socio-emotional wealth (SEW) and evolutionary psychology perspective. Specifically, this study focused on the effect of clan culture. In Chinese firms, clan culture embodies the prominent features of traditional Chinese culture. Therefore, exploring the influence of traditional values such as clan culture can capture the logical basis and cultural foundation for behaviors and motivations of family firms. Moreover, clan culture has been generated, preserved, and refined through a long tradition of common living and production. Moral beliefs have originated from common practice and been passed down through generations, providing a continuous and stable perspective for this study to investigate the effect of regional culture. Focusing on this goal, firstly, the paper reviews the existing literature in the field of clan culture and family business and proposes research hypotheses accordingly. Further, it explains the research methodology of this study, including sample and data sources, variable definition and measurement. On this basis, it analyzes the statistical relationships between variables to verify the hypotheses proposed in this study. Finally, research conclusions are drawn and discussed based on the statistical results.

This study contributes to existing literature in the following two aspects: First, existing research often regards family firms as homogeneous groups, and explores the differences between family firms and nonfamily firms or the evolution process of family firms in different stage. However, this paper, based on the perspective of regional culture, proves that there are also differences among family firms, extending existing research on the family business from the level of “family firm vs. nonfamily firm” to “family firms in different regions.” Second, based on the perspective of SEW and evolutionary psychology, this paper focuses on the impact of regional clan culture on family firms. Existing studies often look for factors that can influence the operation of the family firms based on firm level and family level, while this study focuses on the regional culture, and introduces the macro-level factor into the analysis framework of which factors affect the operation of family firms.

## Literature review and hypotheses

### Clan culture and family management involvement

Previous studies on family businesses suggest that family firms differ from nonfamily firms in that family members with kinship ties participate in business operation in the former (Cui et al., 2018). Therefore, for a firm with two organizational systems (i.e., family and business), factors such as family patterns and philosophy often become the primary reference point of behaviors and decision-making (Souder et al., 2017). Specifically, compared with nonfamily firms, family firms are more vulnerable to the influence of individual needs and emotions; thus, family firms depend on both business and family logic for operational and strategic decisions (Van Gils et al., 2019). Recent research begins to pay attention to the influence of evolutionary psychology on family firm operation (Yu et al., 2020). Evolutionary psychology originated from Darwin’s theory of evolution, which pointed out that, including human beings, the primary goal of animals is to ensure that their genes can be passed on and continued (Lewis et al., 2017; Saad, 2017). During this process, the psychology of the individual will also undergo corresponding changes, showing extra care and love for relatives with genetic similarity, that is, showing altruistic behaviors (Sharma et al., 2020). Likewise, during the operation of family firms, family members will also endeavor to satisfy their own interests in the first priority (Boellis et al., 2016). In other words, family firms carrying families’ efforts and expectations often emphasize the pursuit of special operation goals. Furthermore, owing to blood or marriage ties, family members are usually close, and their interests are consistent. Therefore, family members often actively participate in the business operation to maintain control over the firm and help pursue the family’s goals (Casillas et al., 2019). Strong kinship bonds underpin mutual trust and care among family members (Lewis et al., 2017), and a family firm naturally becomes an important place for family members to communicate and help each other. Therefore, to be involved in the management of firms is often beneficial to family members’ emotional satisfaction in terms of sense of belonging, security, identification, and kindness. Therefore, driven by the blood complex and familial feelings, family members are usually more willing to serve as executives within the family business, that is, to maintain a high level of family involvement in management (Madison et al., 2021).

However, owing to different regional cultures, family members’ levels of motivation to participate in business operations are not identical. As mentioned earlier, clan culture has a profound and lasting influence on local individuals, and it is an important perspective and component of regional culture. Clan culture advocates “glorifying the ancestors,” and local individuals often follow the value of “family interests first” (Zhang, 2020). Influenced by clan culture, individuals are not only willing to strive to uphold family honor but also think of “feeding back” the family. In ancient Chinese, where the imperial examination system was followed, in an effort to contribute to the clan, individuals usually returned to their hometown to worship their ancestors after nomination. They donated funds or purchased lands to assist family members in need after an official promotion. Within the family, children are considered as the continuation of life, and siblings are trusted for positive help. Family members always support each other because blood is thicker than water (Vidacs, 2018). Such natural trust and love among family members from birth is gradually growing; clan norms and moral rules are followed in the long run and in everyday life and production. Further, they form a social trust structure of “internal and external differences pattern” (Cherchem, 2017). It affects the distance in interpersonal relationships and influences the personnel policy of family business. A strong clan culture that emphasizes family bonding may require individuals to reach a consensus on preserving family interests and caring for relatives. From another perspective, as mentioned above, evolutionary psychology emphasizes that on account of the bond derived from genetic similarity, the instinct to care about each other mutually exists among family members. If a region attaches great importance to clan culture, then the connection among family members will be tighter, and the instinctive altruistic tendencies will be more fully displayed and embodied. Therefore, faced with the operation of a family business, individuals have a strong sense of family affection and are willing to believe in the natural emotional connection between family members (Cannella et al., 2015). As a family member, they prefer to use their own resources to serve the family business and share common interests (Miller et al., 2011). Therefore, we hypothesize the following:

H1: Family firms in regions with strong clan culture are more inclined to include family members in management than those in regions with weak clan culture.

### Clan culture and family executive compensation

Existing studies have proposed that in the process of running a family business, family members will not only focus on the acquisition of economic benefits, but also on the enhancement of family prestige (Deephouse and Jaskiewicz, 2013), the expansion of social network (Zahra, 2010) and other non-economic goals such as the realization of succession (Zellweger et al., 2013; Shi et al., 2022), that is, the so-called “socio-emotional wealth” (Gomez-Mejia et al., 2007). In the eyes of family members, a family business is not only a place of work, but also a symbol of family status and glory. As part of a family, family members can often gain a higher social status in the process of working for the family business and share the honor and prestige from the family (Madison et al., 2021). It is precisely because of this spiritual satisfaction that existing studies generally believe family members who work for family firms usually do not ask for high economic returns (Combs et al., 2010). On the other hand, when working for a family business, family members generally believe that they are working for their own family, and their efforts and achievements will ultimately be reflected in their own family business (Pan et al., 2018). According to the above introduction of evolutionary psychology, humans usually have the motivation to help and care for other family members with kinship, and working for family firms can satisfy this complex. In this case, the need for family members to obtain high economic returns from the family business is also reduced.

However, under the influence of different regional cultures, it appears that not all family firms prioritize family’s SEW equally (Zhu et al., 2022). Based on the connection of blood and marriage ties, individuals’ behaviors and motives are more consistent with the family’s interests in regions with strong clan culture. They prefer family noneconomic goals, such as preserving good social reputation, maintaining stable group order, and consolidating harmonious personal relationships (Zellweger et al., 2013). It seems even natural for them to be loyal to the family. As the hallmarks of clan culture, “establishing ancestral hall, constructing genealogy, and setting up clan farmland” all reflect a strong family concept. After living together for long, family members forge an interdependent relationship in which everybody wins or loses together (Wang et al., 2022). Therefore, a family life of dependence and support deepens the idea of mutual help among family members, which is emphasized by evolutionary psychology and forms the attitude of “selfless dedication” (Yu et al., 2020). Applying this logic to family firms’ executive compensation design, those in region with strong clan culture are more likely to pay less to family executives. Given that individuals shaped by a strong clan culture have a deeper sense of family emotion and dedication; family executives could resist utilitarian temptations and pay less attention to the pursuit of individual economic returns. Therefore, we hypothesize the following:

H2: Family firms in regions with strong clan culture pay less to family executives than those in regions with weak clan culture.

### Moderating effect of financial risk

The analysis framework of SEW suggests that the gain and loss of family’s SEW are considered the basic reference point for decision-making and behavior in family firms, and the enterprise’s financial status will affect the prioritization of the family’s SEW goals (Gomez-Mejia et al., 2018). Therefore, higher financial risk will increase differences in motivation levels regarding involvement in family management in different regions. As mentioned earlier, regional clan culture can affect the level of involvement in family management because people hold different attitudes toward the maintenance of family interests and the trust of family relations generated from evolutionary psychology. Specifically, family firms emphasize the pursuit of family’s SEW when the financial situation of the enterprise is good. Thus, family members actively participate in the operation of family firms to maintain control over the firm (Berrone et al., 2012). However, if there is a high financial risk, family firms in regions with weak clan culture may seek to improve their financial situation and turn to pursue economic interests (Alessandri et al., 2018) rather than emphasize the maintenance of family control and preservation of SEW. In contrast, under the influence of a strong clan culture, risks and losses are tolerated by family executives who believe in the cohesion and unity of family members, adhere to the sustainability and development of family firms, and maintain the enthusiasm to participate in business operations. Specifically, for the sake of kinship ties, family members prefer to continue to run a business in poor condition rather than ignore it (Casillas et al., 2019). The worse the financial situation of a family firm, the more prominent the family concept is in individual’s mind and the greater the difference in family management involvement under the influence of different regional cultures. Therefore, we hypothesize the following:

H3: Financial risk will moderate the relationship between clan culture and family management involvement. Specifically, the positive relationship between clan culture and family management involvement will be stronger when the financial risk is high.

Although a poor financial situation of an enterprise may decrease the level of executive compensation of all family firms, it can further stimulate family executives’ loyalty and dedication. Therefore, for a family firm in regions with strong clan culture, it still cannot prevent a family executive from being loyal to the family and working for the firm even if the salary is low. Therefore, some studies have found that family members tend to “indulge” in maintaining the development of family business at all costs (Chirico et al., 2020). They not only do not seek individual economic returns but also donate private funds to help the family firm overcome difficulties. According to this logic, when enterprises are faced with higher financial risks, family executives who do not prioritize kinship ties are unable to tolerate economic losses and thus turn to pursue improvement of the financial situation (Alessandri et al., 2018). However, in the regions deeply influenced by clan culture, individuals always maintain the greatest concern for family’s SEW, further reducing the requirements in terms of salaries, which leads to a gradual increase in the differences in the family executive compensation under different cultures. The higher the financial risk of family firms, the stronger the family cohesion and loyalty are motivated and the greater the difference in effect under different regional cultures. Therefore, we hypothesize the following:

H4: Financial risk will moderate the relationship between clan culture and family executive compensation. Specifically, the negative relationship between clan culture and family executive compensation will be stronger when the financial risk is high.

## Materials and methods

### Sample and data source

The study sample includes the data on all family firms listed on the Small & Medium Enterprise Board and Growth Enterprise Board of the China Shenzhen Stock Exchange because a several family firms listed on the Main Board are state-owned or have a considerable government background, while the business operations of firms on the Small & Medium Enterprise Board and Growth Enterprise Board are less affected by the government (Yu et al., 2020). Therefore, using data from Small & Medium Enterprise Board and Growth Enterprise Board is more suitable to achieve the purpose of this study.

Consistent with earlier studies (Gomez-Mejia et al., 2018; Kotlar et al., 2018), we define a “family firm” in this study if it meets the following three conditions: (a) the chairperson is the actual controller of the firm; (b) the family owns at least 20% of firm shares; and (c) besides the chairperson, at least one additional family member participates in the business operation. If the family member holds the position in the TMT, it is considered that she or he participates in the business operation of family firm as a “family executive.” The personal information of the chairperson and position information of the relatives should be included in the prospectus and the information of executives who join or leave the firm every year should be released in the annual reports. Therefore, we manually collected the data from the prospectus and annual reports, which resulted in a final sample of 625 family firms.

In this study, the genealogy data of cities were obtained by consulting The General Catalog of Chinese Genealogy published by Shanghai Ancient Books Publishing House. There are more than 50,000 family history records involving 608 surnames. The main body of the general catalog is in the order of surname strokes. The first line of each genealogy record contains a county name listed in a square bracket to show the region that the genealogy belongs to. Therefore, this study extracted the county names of genealogies in the square brackets for all genealogy records to obtain a list of regions corresponding to the general catalog. For example, starting from the surname “Ding” (“丁”) with two strokes in the text, the first record is a genealogy for the Ding family numbered 001–0001 located in “Yanshan, Hebei” (“河北盐山”). In this study, the county name “Yanshan, Hebei” (“河北盐山”) was extracted to the list of genealogy regions. For the second record, the genealogy of Ding family numbered 001–0002, the county name “Chongming, Shanghai” (“上海崇明”) was extracted from it. Similarly, the county names of all records were extracted to the list of genealogy regions according to the order. Based on the list of genealogy regions, this study further counted the frequency of each “county name” to calculate the number of genealogy volumes belonging to the region. Finally, the number of genealogy volumes at the prefecture level was obtained by manual calculation. To standardize the genealogy data with the regional population, this study obtained demographic data of each city by consulting the statistical yearbooks.

Financial status, governance structure information, and personal information of the chairperson used in this study were all obtained from the China Stock Market & Accounting Research (CSMAR) database. Because the Small & Medium Enterprise Board and Growth Enterprise Board were established in 2004 and 2009, respectively, the study period was 2004–2017. To avoid the impact of outliers on the study results, we excluded 12 observations from the sample because the average salary of family executives is less than that of ordinary employees. Therefore, this study finally obtained a panel dataset consisting of 625 family firms and 3,126 observations.

### Variable definition and measurement

#### Family management involvement

Consistent with previous research, we determine a family executive if the family member serves as a TMT member. This study measures family management involvement as the relative proportion of family executives on the TMT in family firms. As the composition of TMT is likely to change within 1 year, this study codes the status of family executive and TMT based on the position information at the end of the fiscal year disclosed in the annual report. It is presented in the form of percentage. The mean value of family executive proportion is 15.823, which means that the number of family executives accounts for 15.823% of the total number of TMT on average. This study also uses the absolute number of family executives on the TMT to measure family management involvement for a robustness test.

#### Family executive compensation

According to the existing research, we develop two measurements for family executive compensation: relative and absolute (Cathcart et al., 2020). The relative compensation of family executives is calculated by the gap of the average salary between family executives and ordinary employees, which represents the level of family executive compensation relative to ordinary employees. We also use the natural logarithm of the average of family executive salaries to measure the absolute level of family executive compensation.

#### Clan culture

This study measures the strength of clan culture through the number of genealogy per 10,000 people in the region where family firms are located (Zhang, 2019). In Chinese culture, genealogy records the names, dates of birth, blood relations, and marriage information of all family members in the family from ancestors to the present, marking the position of each family member in the family. Through the genealogy, family members can understand the family, especially the ancestors, who are other members of the same family as themselves, and more importantly, what kind of connections and ties they have with the family. In other words, a genealogy is a “literal bridge” that connects each family member. If the number of existing genealogy in the area is more, it shows that the local people attach greater importance to the family, and hope that they can clarify their relationship with the family and understand who is their “own family” by preserving the genealogy. Because of this, the more genealogy that exists in the region, the more it can indicate the prevalence of clan culture in the region (Xiong et al., 2021). On the contrary, if the genealogy is lost or not passed down from generation to generation, it means that people do not care much about the clan to a certain extent, or the clan does not play an important role in people’s daily life, which can indicate that the cultural influence of the clan in the region is limited. In view of the above, this study measures the concept of “clan culture” by dividing the number of existing genealogies at the location of family firms by the number of local populations.

#### Financial risk

As mentioned earlier, family firms vary in their prioritization of SEW and financial goals. In addition to the firm’s regional cultural environment, the internal financial situation may shape a family firm’s prioritization of SEW, which leads to different characteristics of firm behavior. Therefore, this study chooses corporate financial risk as a research perspective to investigate how it affects a family firm’s prioritization of the pursuit of SEW under different regional cultures. Studies have shown that the higher the firm’s financial leverage measured by the book values of total liabilities divided by total assets, the greater the firm’s operating pressure and financial risk (Miller et al., 2013; Hennart et al., 2019). Therefore, this study uses financial leverage as a proxy for the financial risk of family firms. The mean value of financial risk is 0.316, indicating that the average level of a firm’s financial leverage is 31.6%.

#### Control variables

In this study, the number of employees and firm age are used to control for the impact of firm size on the results (Zulfiqar et al., 2022). The unit of firm age is “year,” and the mean value of 11.575 means that the average age of the family firms in the sample is 11.575 years. To address the issue of business operating situation, we control for the return on assets (ROA) and cash ratio (Qi et al., 2022). ROA is presented in decimal form, with an average value of 0.053, indicating that the average ROA of the family firms in the sample is 5.3%. We also control for the board independence, measured by the ratio of the number of independent directors to the total number of directors. Board independence is also presented in decimal form, with an average value of 0.377, indicating that the average proportion of the family firms’ independent directors in the sample is 37.7%. As the personal characteristics of the chairperson may affect the results, chairperson’s age and gender (male = 1) are included as control variables. Given that equity incentive and executive’s human capital may affect compensation incentive, we control for the average ownership and average education level of family executives. Education level of family member is measured as follows: “1″ if the member graduated from a secondary school, “2″ if graduated from college, “3″ if held a bachelor’s degree, “4″ if held a master’s degree, and “5″ if held a doctor degree. At the regional level, we also control for variables such as regional market index and population size. Additionally, we control for year and industry.

## Results

### Descriptive statistics and correlations

The descriptive statistics and correlations of the dependent, independent, and control variables are presented in Table 1.

**Table 1:** Results of descriptive statistics and correlations.

	Variable	Mean	SD	1	2	3	4	5	6	7	8	9	10	11	12	13	14
1	Family executive relative proportion	15.823	5.618														
2	Family executive relative compensation	46.626	42.292	**−0.085**													
3	Clan culture	1.146	1.649	**0.088**	**−0.038**												
4	Financial risk	0.316	0.173	**−0.117**	**0.149**	−0.016											
5	Employee number	7.218	0.993	**−0.038**	**0.423**	−0.021	**0.405**										
6	Firm age	11.575	5.173	0.033	**0.162**	0.022	0.024	**0.036**									
7	ROA	0.053	0.048	**−0.066**	**0.108**	−0.007	**−0.329**	0.008	−0.020								
8	Cash ratio	1.860	5.362	0.033	0.005	**−0.060**	**−0.360**	**−0.195**	**−0.083**	**0.111**							
9	Board independence	0.377	0.055	0.019	**0.079**	−0.015	0.000	0.009	−0.007	0.006	−0.009						
10	Chairperson age	52.443	8.513	**0.076**	**0.042**	0.003	**−0.066**	**−0.039**	**0.169**	−0.022	−0.025	**−0.095**					
11	Chairperson gender	0.930	0.255	**−0.059**	0.012	−0.003	0.002	0.034	−0.001	0.009	0.018	**−0.036**	**0.078**				
12	Family executive ownership	18.021	4.285	**0.065**	**0.035**	0.055	**−0.042**	−0.015	**−0.104**	**0.181**	**0.099**	**0.127**	**−0.102**	−0.011			
13	Family executive education	3.232	3.167	**−0.055**	**0.132**	**−0.104**	**0.035**	**0.089**	−0.011	0.007	0.023	**0.072**	−0.010	**−0.070**	−0.004		
14	Regional market index	8.554	1.498	0.022	**0.172**	0.237	**0.068**	**0.048**	**0.339**	0.021	**−0.113**	**0.057**	0.003	0.004	**−0.057**	0.012	
15	Regional population size	8.775	0.521	0.012	**0.050**	−0.061	**0.088**	**0.163**	**0.071**	−0.013	**−0.049**	−0.008	0.015	**0.067**	−0.018	**0.042**	**0.122**

To facilitate the analysis, family executive relative proportion and family executive ownership are presented in percentages. We take the natural logarithm of employee number and regional population size (in 10,000 people). The unit of family executive relative compensation is 10,000 RMB yuan.

Values in bold are significant at *p* < 0.05.

### Hypotheses testing

On the basis of the correlation analysis, we employ the regression method for further testing the research hypotheses. The results of testing Hypothesis 1, that is, the relationship between clan culture and family management involvement, are shown in Table 2. Models 1 and 3 in Table 2 show the control models. Model 2 in Table 2 shows that when the dependent variable family management involvement is measured by family executive relative proportion, clan culture has a significant positive effect on family management involvement (*b* = 0.182, *p* < 0.01). While Model 4 in Table 2 shows that when the dependent variable family management involvement is measured by the family executive absolute number, clan culture also has a significant positive effect on family management involvement (*b* = 0.013, *p* < 0.1). Therefore, the results suggest that family firms in regions with a strong clan culture are more inclined to include family members in the top management than those in regions with weak clan culture, indicating support for Hypothesis 1.

**Table 2:** Effect of clan culture on family management involvement.

	Family executive relative proportion		Family executive absolute number	
Variable	Model 1	Model 2	Model 3	Model 4
Clan culture		0.182^**^		0.013+
		(0.064)		(0.007)
Employee number	−0.187+	−0.183+	0.023+	0.023+
	(0.106)	(0.106)	(0.012)	(0.012)
Firm age	0.035	0.036	0.001	0.001
	(0.023)	(0.023)	(0.003)	(0.003)
ROA	−8.722^***^	−8.556^***^	−0.300	−0.288
	(2.135)	(2.134)	(0.232)	(0.232)
Cash ratio	0.036+	0.039^*^	0.003	0.003
	(0.019)	(0.019)	(0.002)	(0.002)
Board independence	1.965	2.101	−0.879^***^	−0.867^***^
	(1.833)	(1.831)	(0.208)	(0.208)
Chairperson age	0.055^***^	0.054^***^	0.005^***^	0.005^***^
	(0.012)	(0.012)	(0.001)	(0.001)
Chairperson gender	−1.814^***^	−1.783^***^	−0.079+	−0.077+
	(0.393)	(0.393)	(0.043)	(0.043)
Family executive ownership	0.099^***^	0.096^***^	0.005+	0.004+
	(0.025)	(0.025)	(0.003)	(0.003)
Family executive education	−0.293^*^	−0.261+	−0.022	−0.020
	(0.144)	(0.144)	(0.016)	(0.016)
Regional market index	0.236^**^	0.166+	0.001	−0.004
	(0.081)	(0.085)	(0.009)	(0.009)
Regional population size	0.076	0.129	0.004	0.008
	(0.195)	(0.196)	(0.022)	(0.022)
Year	Control	Control	Control	Control
Industry	Control	Control	Control	Control
N	3,126	3,126	3,126	3,126
*R*^2^/Pseudo *R*^2^	0.060	0.063	0.008	0.008
F/Chi^2^	5.64	5.72	75.24	78.70

Standard errors are in parentheses. ^+^*p* < 0.1, ^*^p < 0.05, ^**^p < 0.01, ^***^p < 0.001.

Hypothesis 2 proposes that family firms in regions with strong clan culture pay less compensation to family executives than those in regions with weak clan culture. Table 3 shows the results. Models 1 and 3 in Table 3 include only the control variables. Model 2 in Table 3 shows that when the dependent variable family executive compensation is measured by the family executive relative compensation, there is a significant negative effect of clan culture on family executive compensation (*b* = −1.215, *p* < 0.01). While Model 4 in Table 3 shows that when the dependent variable family executive compensation is measured by the family executive absolute compensation, there is a significant negative effect of clan culture on family executive compensation (*b* = −0.027, *p* < 0.001). Therefore, the results suggest that family executives are less compensated in family firms in regions with strong clan culture than those in regions with weak clan culture, which empirically supports Hypothesis 2.

**Table 3:** Effect of clan culture on family executive compensation.

	Family executive relative compensation		Family executive absolute compensation	
Variable	Model 1	Model 2	Model 3	Model 4
Clan culture		−1.215^**^		−0.027^***^
		(0.424)		(0.006)
Employee number	18.842^***^	18.811^***^	0.232^***^	0.231^***^
	(0.700)	(0.699)	(0.010)	(0.010)
Firm age	0.593^***^	0.586^***^	0.010^***^	0.009^***^
	(0.150)	(0.150)	(0.002)	(0.002)
ROA	83.053^***^	81.947^***^	1.900^***^	1.876^***^
	(14.081)	(14.070)	(0.199)	(0.199)
Cash ratio	0.820^***^	0.797^***^	0.007^***^	0.007^***^
	(0.127)	(0.128)	(0.002)	(0.002)
Board independence	46.162^***^	45.254^***^	0.417^*^	0.397^*^
	(12.085)	(12.075)	(0.171)	(0.171)
Chairperson age	0.226^**^	0.233^**^	0.004^***^	0.005^***^
	(0.080)	(0.080)	(0.001)	(0.001)
Chairperson gender	−0.327	−0.535	0.066+	0.062+
	(2.593)	(2.591)	(0.037)	(0.037)
Family executive ownership	0.334^*^	0.358^*^	0.003	0.004
	(0.162)	(0.162)	(0.002)	(0.002)
Family executive education	4.584^***^	4.369^***^	0.104^***^	0.099^***^
	(0.950)	(0.952)	(0.013)	(0.013)
Regional market index	2.733^***^	3.202^***^	0.081^***^	0.091^***^
	(0.537)	(0.560)	(0.008)	(0.008)
Regional population size	−3.139^*^	−3.498^**^	−0.054^**^	−0.062^***^
	(1.289)	(1.294)	(0.018)	(0.018)
Year	Control	Control	Control	Control
Industry	Control	Control	Control	Control
*N*	3,126	3,126	3,126	3,126
*R* ^2^	0.278	0.280	0.340	0.344
*F*	34.04	33.40	45.52	45.07

Standard errors are in parentheses. ^+^*p* < 0.1, ^*^p < 0.05, ^**^p < 0.01, ^***^p < 0.001.

Hypotheses 3 and 4 predict that financial risk may moderate the effects of clan culture. Therefore, financial risk is added to the models as a moderating variable. The test results are shown in Table 4.

**Table 4:** The moderating effect of financial risk.

	Family executive relative proportion		Family executive absolute number		Family executive relative compensation		Family executive absolute compensation	
Variable	Model 1	Model 2	Model 3	Model 4	Model 5	Model 6	Model 7	Model 8
Clan culture ^*^ Financial risk		1.323^***^		0.068+		−6.713^**^		−0.075^*^
		(0.369)		(0.040)		(2.448)		(0.035)
Financial risk	−4.684^***^	−6.276^***^	−0.286^***^	−0.371^***^	3.777	11.854^*^	−0.004	0.086
	(0.745)	(0.866)	(0.084)	(0.098)	(4.947)	(5.752)	(0.070)	(0.081)
Clan culture	0.177^**^	−0.225+	0.013+	−0.008	−1.210^**^	0.827	−0.027^***^	−0.004
	(0.064)	(0.129)	(0.007)	(0.014)	(0.424)	(0.855)	(0.006)	(0.012)
Employee number	0.119	0.133	0.042^**^	0.043^**^	18.568^***^	18.492^***^	0.232^***^	0.231^***^
	(0.116)	(0.116)	(0.013)	(0.013)	(0.768)	(0.767)	(0.011)	(0.011)
Firm age	0.037	0.036	0.001	0.001	0.585^***^	0.589^***^	0.009^***^	0.009^***^
	(0.023)	(0.023)	(0.003)	(0.003)	(0.150)	(0.150)	(0.002)	(0.002)
ROA	−13.757^***^	−14.009^***^	−0.600^*^	−0.612^*^	86.140^***^	87.423^***^	1.872^***^	1.886^***^
	(2.276)	(2.273)	(0.247)	(0.247)	(15.105)	(15.096)	(0.213)	(0.213)
Cash ratio	0.004	−0.002	0.001	0.001	0.825^***^	0.854^***^	0.007^***^	0.007^***^
	(0.020)	(0.020)	(0.002)	(0.002)	(0.133)	(0.133)	(0.002)	(0.002)
Board independence	2.027	1.974	−0.869^***^	−0.872^***^	45.314^***^	45.585^***^	0.397^*^	0.400^*^
	(1.820)	(1.816)	(0.208)	(0.208)	(12.076)	(12.064)	(0.171)	(0.170)
Chairperson age	0.048^***^	0.047^***^	0.005^***^	0.004^***^	0.238^**^	0.246^**^	0.005^***^	0.005^***^
	(0.012)	(0.012)	(0.001)	(0.001)	(0.080)	(0.080)	(0.001)	(0.001)
Chairperson gender	−1.781^***^	−1.723^***^	−0.078+	−0.075+	−0.537	−0.828	0.062+	0.058
	(0.390)	(0.390)	(0.043)	(0.043)	(2.591)	(2.591)	(0.037)	(0.037)
Family executive ownership	0.097^***^	0.098^***^	0.005+	0.005+	0.358^*^	0.351^*^	0.004	0.004
	(0.024)	(0.024)	(0.003)	(0.003)	(0.163)	(0.162)	(0.002)	(0.002)
Family executive education	−0.280+	−0.284^*^	−0.021	−0.022	4.384^***^	4.404^***^	0.099^***^	0.100^***^
	(0.143)	(0.143)	(0.016)	(0.016)	(0.952)	(0.951)	(0.013)	(0.013)
Regional market index	0.186^*^	0.182^*^	−0.003	−0.003	3.186^***^	3.204^***^	0.091^***^	0.091^***^
	(0.084)	(0.084)	(0.009)	(0.009)	(0.561)	(0.560)	(0.008)	(0.008)
Regional population size	0.146	0.185	0.009	0.012	−3.511^**^	−3.709^**^	−0.062^***^	−0.064^***^
	(0.195)	(0.195)	(0.022)	(0.022)	(1.294)	(1.295)	(0.018)	(0.018)
Year	Control	Control	Control	Control	Control	Control	Control	Control
Industry	Control	Control	Control	Control	Control	Control	Control	Control
*N*	3,126	3,126	3,126	3,126	3,126	3,126	3,126	3,126
*R*^2^/Pseudo *R*^2^	0.074	0.078	0.009	0.010	0.280	0.282	0.344	0.345
*F*/Chi^2^	6.71	6.89	90.51	93.34	32.51	31.92	43.84	42.86

Standard errors are in parentheses. ^+^*p* < 0.1, ^*^p < 0.05, ^**^p < 0.01, ^***^p < 0.001.

Models 2 and 4 in Table 4 show that the interaction of clan culture and financial risk has positive and significant effects on family executive relative proportion (*b* = 1.323, p < 0.001) and family executive absolute number (*b* = 0.068, *p* < 0.1), indicating support for Hypothesis 3, that is, the positive relationship between clan culture and family management involvement will be stronger when the financial risk is high.

With regard to Hypothesis 4, Models 6 and 8 in Table 4 show that the interaction of clan culture and financial risk has negative and significant effects on family executive relative compensation (*b* = −6.713, p < 0.01) and family executive absolute compensation (*b* = −0.075, *p* < 0.05), indicating that the negative relationship between clan culture and family executive compensation will be stronger when the financial risk is high. Therefore, Hypothesis 4 is supported.

**Figure 1:**
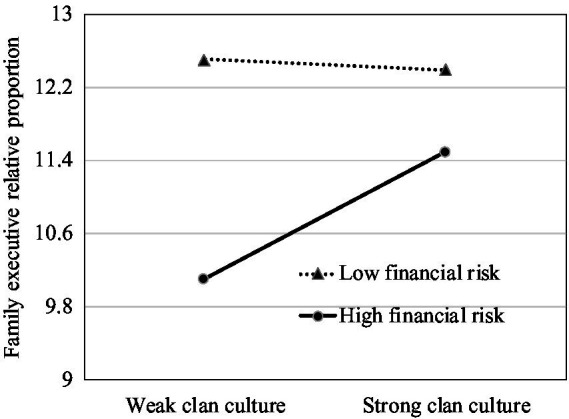
Clan culture, family executive relative proportion, and financial risk.

**Figure 2:**
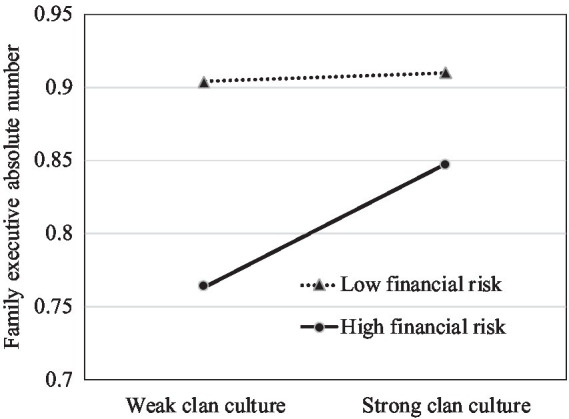
Clan culture, family executive absolute number, and financial risk.

**Figure 3:**
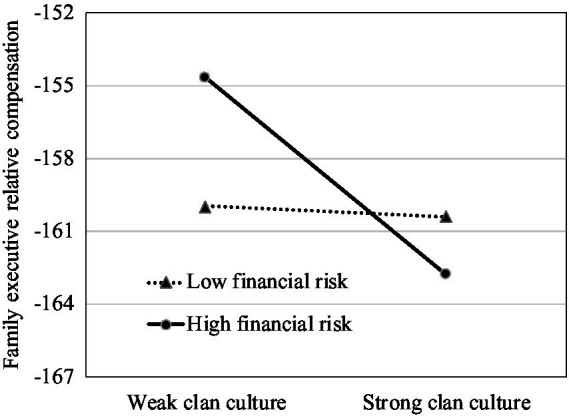
Clan culture, family executive relative compensation, and financial risk.

**Figure 4:**
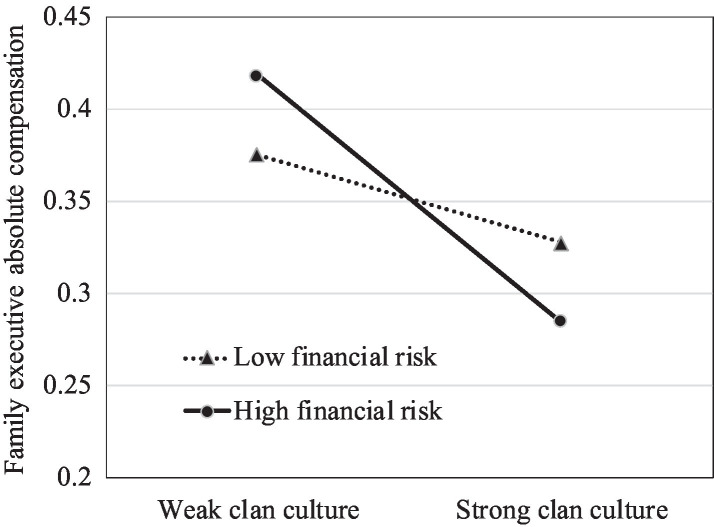
Clan culture, family executive absolute compensation, and financial risk.

To further explore the moderating effect of financial risk, we plotted the impact of clan culture on family management involvement and executive compensation for different levels of the firm’s financial risk. We set the low-level group of financial risk as below the mean value and the high-level group as above the mean value. Meanwhile, we divided clan culture into two groups by its mean value. Consistent with Hypothesis 3, Figures 1, 2 show stronger positive relationships between clan culture and family management involvement when the financial risk is high than when it is low. As demonstrated in Figures 3, 4, the negative effects of clan culture on family executive compensation are stronger when the financial risk is high than when it is low, thus supporting Hypothesis 4.

### Robustness test

To ensure the robustness of the research results, two methods have been used to measure the dependent variables in the regression analysis, and the results can support the hypotheses of this study. Accordingly, the following robustness tests are performed.

To exclude the interference of the definition of family firm to the results, this study redefines family firm according to different criteria. Previous studies have usually considered the ownership of family members at least 20% as one of the criteria to judge whether the firm belongs to the family business group. This study further takes the family ownership at least 10, 15, and 25% as the criteria to define the family firm. Regression analysis is conducted based on the reselected samples, and the results are still consistent with the original results. Owing to space limitations, this study only lists the regression results when the criteria for the definition of family firm are “family ownership not less than 25%” (Table 5).

**Table 5:** Robustness test results of redefining family firm with family ownership no less than 25%.

	Family executive relative proportion		Family executive relative compensation	
	Model 1	Model 2	Model3	Model 4
Clan culture ^*^ Financial risk		1.419^***^		−6.803^**^
		(0.373)		(2.468)
Financial risk		−6.364^***^		11.737^*^
		(0.873)		(5.783)
Clan culture	0.194^**^	−0.243+	−1.264^**^	0.797
	(0.065)	(0.130)	(0.426)	(0.858)
Employee number	−0.202+	0.113	18.514^***^	18.204^***^
	(0.107)	(0.116)	(0.702)	(0.771)
Firm age	0.038+	0.038+	0.602^***^	0.605^***^
	(0.023)	(0.023)	(0.150)	(0.150)
ROA	−8.629^***^	−14.027^***^	80.646^***^	85.961^***^
	(2.142)	(2.280)	(14.083)	(15.099)
Cash ratio	0.038+	−0.003	0.785^***^	0.840^***^
	(0.019)	(0.020)	(0.127)	(0.133)
Board independence	2.173	2.093	43.362^***^	43.574^***^
	(1.846)	(1.830)	(12.132)	(12.120)
Chairperson age	0.054^***^	0.046^***^	0.235^**^	0.247^**^
	(0.012)	(0.012)	(0.080)	(0.080)
Chairperson gender	−1.887^***^	−1.835^***^	0.494	0.201
	(0.397)	(0.394)	(2.609)	(2.609)
Family executive ownership	0.087^***^	0.088^***^	0.477^**^	0.475^**^
	(0.026)	(0.026)	(0.171)	(0.171)
Family executive education	−0.296^*^	−0.320^*^	4.461^***^	4.504^***^
	(0.145)	(0.144)	(0.954)	(0.953)
Regional market index	0.148+	0.166+	3.321^***^	3.329^***^
	(0.086)	(0.086)	(0.567)	(0.567)
Regional population size	0.085	0.136	−3.520^**^	−3.723^**^
	(0.199)	(0.197)	(1.307)	(1.307)
Year	Control	Control	Control	Control
Industry	Control	Control	Control	Control
*N*	3,079	3,079	3,079	3,079
*R^2^*	0.064	0.080	0.278	0.280
*F*	5.79	6.97	32.52	31.08

Standard errors are in parentheses. ^+^*p* < 0.1, ^*^p < 0.05, ^**^p < 0.01, ^***^p < 0.001.

Clan culture is more prosperous in southern China than in northern China and stronger in eastern China than in western China. To further prove that the variations in TMT characteristics in family firms are not only caused by differences in geographical location, this study conducts a robustness test. We select the subsamples of family firms from Jiangsu, Zhejiang, and Shanghai regions. The three regions belong to eastern China, so there are no so-called geographical differences. However, in these three regions, there is a prevailing trend of revising genealogy in some cities, while the genealogy in other cities is rare, which provides a good research object for this study. Table 6 shows the results based on this subsample. The results show that regional clan culture rather than geographical location leads to differences in the TMT characteristics.

**Table 6:** Robustness test results based on subsamples of Jiangsu, Zhejiang, and Shanghai regions.

	Family executive relative proportion		Family executive relative compensation	
	Model 1	Model 2	Model3	Model 4
Clan culture ^*^ Financial risk		1.863^***^		−8.468^**^
		(0.551)		(2.913)
Financial risk		−7.592^***^		21.421^*^
		(1.997)		(10.561)
Clan culture	0.286^***^	−0.263	−1.164^**^	1.292
	(0.085)	(0.181)	(0.449)	(0.957)
Employee number	0.206	0.377	18.577^***^	18.510^***^
	(0.221)	(0.233)	(1.165)	(1.230)
Firm age	0.040	0.036	0.986^***^	1.006^***^
	(0.040)	(0.040)	(0.211)	(0.210)
ROA	−11.937^***^	−15.567^***^	65.072^***^	69.329^***^
	(3.569)	(3.794)	(18.815)	(20.063)
Cash ratio	0.098	0.013	1.357^**^	1.396^**^
	(0.084)	(0.091)	(0.441)	(0.482)
Board independence	5.665	5.692	22.816	23.805
	(3.525)	(3.506)	(18.582)	(18.540)
Chairperson age	0.109^***^	0.101^***^	0.561^***^	0.586^***^
	(0.020)	(0.020)	(0.108)	(0.108)
Chairperson gender	−2.006^**^	−1.918^**^	−3.428	−4.136
	(0.694)	(0.692)	(3.657)	(3.658)
Family executive ownership	0.051	0.051	0.258	0.277
	(0.043)	(0.043)	(0.229)	(0.229)
Family executive education	−0.656^*^	−0.662^**^	4.923^***^	5.027^***^
	(0.256)	(0.255)	(1.352)	(1.349)
Regional market index	−0.697	−0.782	2.118	2.271
	(0.483)	(0.480)	(2.545)	(2.541)
Regional population size	−2.170^***^	−2.017^***^	−7.389^**^	−7.886^**^
	(0.516)	(0.514)	(2.718)	(2.718)
Year	Control	Control	Control	Control
Industry	Control	Control	Control	Control
*N*	1,105	1,105	1,105	1,105
*R^2^*	0.108	0.120	0.318	0.323
*F*	3.80	4.06	14.68	14.18

Standard errors are in parentheses. ^+^*p* < 0.1, ^*^p < 0.05, ^**^p < 0.01, ^***^p < 0.001.

Considering the possible non-linear relationships between independent variables and dependent variables, the following robustness tests were carried out in this study. This study first calculated the squared value of the independent variable and performed a regression with the dependent variable. The results show that the correlation coefficient between the squared value of the independent variable and the dependent variable is *b* = −0.005, but the significance level is *p* > 0.1, indicating that there is no statistically significant correlation between the squared value of the independent variable and the dependent variable. That is, there is no U-shaped or inverted U-shaped relationship between the independent variable “family culture” and the dependent variable “family executive involvement.” On this basis, this study further explores whether there is a significant correlation between the cube of the independent variable and the dependent variable. This study calculated the cube of the independent variable and performed a regression with the dependent variable. The results show that the regression coefficient between the cubic value of the independent variable and the dependent variable is *b* = 0.002, and the significance level is *p* > 0.1, indicating that there is no statistically significant correlation between the cubic value of the independent variable and the dependent variable.

Although as mentioned above, family businesses from the SME board and ChiNext board are more suitable for this study because the process of growth and development is less affected by the government. However, considering that there are also a certain number of family businesses among the companies listed on the main board of the Shanghai or Shenzhen stock exchanges, in order to cover more family businesses in the research sample and further improve the rigor and applicability of the conclusions, the family businesses listed on the main board of the Shanghai Stock Exchange and the Shenzhen Stock Exchange were also collected for robustness testing. In the process of screening family businesses listed on the main board, this study also adheres to the following criteria “first, family members are the actual controllers of the enterprise; second, the family shareholding ratio needs to be higher than 20%; third, in addition to the chairman, at least one family member is involved in the operation of the business.” According to this standard, this study obtained a total of 471 family businesses listed on the main board. Based on a research sample consisting of all 1,096 family businesses listed on the main board, SME board and ChiNext board, this study conducts a robustness test. The results show that after including the family businesses listed on the main board, Hypotheses 1–3 are still significant.

## Discussion

The results of this study found that, in areas where clan culture is prevalent, local family firms are indeed more inclined to arrange family members to participate in the business operations, and family members involved in the business are also willing to accept lower remuneration. The reason of this phenomenon is that both family firms and family members involved in business operations pay attention to not only economic returns such as profits and earnings, but also psychological returns such as socio-emotional wealth when making decisions (Lohe et al., 2021; Shi et al., 2022). It has been suggested that the reason why family firms prefer to bring in family members to participate in business operations is to achieve the specific business goals of family firms such as maintaining family control and satisfying altruistic complex (Basco et al., 2019; Casillas et al., 2019). The fact that local family firms in areas where clan culture is prevalent are more willing to bring in family members to participate in business operations is an indication that in that area, people are more focused on psychological returns such as socio-emotional wealth due to the influence of the regional culture. In other words, the regional culture influences the business decisions of family business operators, especially personnel decisions related to how to design the top management team, and inadvertently shapes the characteristics of the local family firm’s top management team. The opinion is also supported by the fact that family members involved in business operations in this region are also willing to accept lower remuneration. The reason why these family members are willing to accept lower remuneration is that they are also satisfied with their psychological dedication to the family in the process of participating in the business operations (Barontini and Bozzi, 2018). Compared with obtaining economic returns, the psychological returns make them feel satisfied equally. The more prevalent the clan culture is, the lower the remuneration that the family members ask for, which also shows that the regional culture can make people pay more attention to obtaining psychological returns and affect the decision-making preference of local people.

Also, this study found that when the financial condition of family business is poor, the impact of regional culture on local family businesses is more pronounced. The existing studies often believe that there is a diminishing marginal effect of economic versus psychological returns for family firms (Miller and Le Breton, 2014), i.e., when family firms have already obtained generous economic returns, they will focus more on acquiring psychological returns, and when economic returns are insufficient, they will temporarily weaken their pursuit of psychological returns and focus on economic returns instead (Chen et al., 2022). The findings of this study, however, indicate that when the financial condition of family business is poor, the differences in the importance attached to psychological returns is more pronounced between family firms in areas where clan culture is prevalent and those in areas where clan culture is indifferent. Family firms in areas where clan culture is prevalent still value psychological returns such as socio-emotional wealth and are not willing to turn to economic returns as family firms in areas where clan culture is indifferent, thus again demonstrating that regional culture can influence the decision-making of local family firms. Compared with previous studies that focused more on the differences in top management team design between family and non-family firms (De Massis et al., 2021; Zhang et al., 2021), this study focuses on the differences between family firms from different regions and under the influence of different regional cultures, proposes hypotheses based on the clan culture perspective and verifies the related logic, which promotes the existing studies to go beyond the family level and the firm level to analyze what factors can influence the operation of family firms at the macro level.

## Conclusion

Based on 625 family firms listed on the Small & Medium Enterprise Board and Growth Enterprise Board of the China Shenzhen Stock Exchange, we find that compared to regions with weak clan culture, family firms in regions with strong clan culture are more inclined to include family members in management. Meanwhile, family executives of family firms in regions with strong clan culture usually get paid less. Specifically, the differences between family firms in regions with strong clan culture and those in regions with weak clan culture will be stronger when the financial risk is high.

### Theoretical implications

This study mainly has two theoretical implications. First, it proves that no family business is a completely homogeneous group, and there are differences among family firms, which extend existing research on family business from the level of “family firm vs. nonfamily firm” to “family firms in different regions.” Studies have regarded family business as a homogeneous whole and focused on the differences between family and nonfamily firms. Based on the perspective of clan culture, this study further proposes and proves that there are also significant differences among family firms affected by different regional cultures. Owing to regional culture, family firms from different regions have different motivation levels and then show different firm characteristics. Therefore, this study takes regional cultural differences as a clue to explain the heterogeneity of family firms and promotes existing studies to focus on what are the differences between family enterprises.

Second, it explores the relationship between regional culture and family firm’s TMT and proves that TMT characteristics in family firms depend not only on micro-level factors such as enterprise and family, but also on macro-level factors such as regional culture. Studies have explored the factors affecting the TMT of family firms based on the enterprise or family level. However, it cannot be ignored that the family business is a special form of business based on the family. It is inevitably affected by the local culture. Therefore, this study proves that regional culture also affects the characteristics of TMT in family firms and introduces the macro-level factor into the analysis framework of “how to design the TMT of family firm.”

### Limitations and future research

This study explores the impact of regional culture on the TMT characteristics of local family firms only from the perspective of clan culture. Future research works should employ other perspectives of regional culture. This study used listed family firms as the research object; future studies can choose an unlisted family firm as the research object to test whether the conclusions of this study are universal. Finally, the development of family members and family firms is probably dependent on multiple regional cultures, so future research can be conducted on the basis of families’ multicultural backgrounds for an in-depth study of the influence of regional cultures on the operation of local family firms.

## Data availability statement

The raw data supporting the conclusions of this article will be made available by the authors, without undue reservation.

## Author contributions

XY and XC: made the theoretical design of the article. YZ and WZ: wrote the manuscript. XY and HL: reviewed and revised the manuscript. YZ, XC, and YC: collected and analysed the data. All authors contributed to the article and approved the submitted version.

## Funding

The author(s) disclosed receipt of the following financial support for the research, authorship, and/or publication of this article: National Natural Science Foundation of China (no. 72002232), Humanity and Social Science Foundation of Ministry of Education of China (no. 19YJC630207), and Outstanding Youth Project of Central University of Finance and Economics (no. QYP202106).

## Conflict of interest

The authors declare that the research was conducted in the absence of any commercial or financial relationships that could be construed as a potential conflict of interest.

## Publisher’s note

All claims expressed in this article are solely those of the authors and do not necessarily represent those of their affiliated organizations, or those of the publisher, the editors and the reviewers. Any product that may be evaluated in this article, or claim that may be made by its manufacturer, is not guaranteed or endorsed by the publisher.
